# TAK1: A Molecular Link Between Liver Inflammation, Fibrosis, Steatosis, and Carcinogenesis

**DOI:** 10.3389/fcell.2021.734749

**Published:** 2021-10-14

**Authors:** Weijun Wang, Wenkang Gao, Qingjing Zhu, Afnan Alasbahi, Ekihiro Seki, Ling Yang

**Affiliations:** ^1^Division of Gastroenterology, Union Hospital, Tongji Medical College, Huazhong University of Science and Technology, Wuhan, China; ^2^Department of Liver Diseases, Wuhan Jinyintan Hospital, Wuhan, China; ^3^Department of Medicine, Cedars-Sinai, Los Angeles, CA, United States

**Keywords:** TAK1, TGF-β signaling, WNT signaling, AMPK signaling, inflammation, liver fibrosis, hepatosteatosis, carcinogenesis

## Abstract

Chronic insult and persistent injury can cause liver inflammation, fibrosis, and carcinogenesis; it can also be associated with metabolic disorders. Identification of critical molecules that link the process of inflammation and carcinogenesis will provide prospective therapeutic targets for liver diseases. Rapid advancements in gene engineering technology have allowed the elucidation of the underlying mechanism of transformation, from inflammation and metabolic disorders to carcinogenesis. Transforming growth factor-β-activated kinase 1 (TAK1) is an upstream intracellular protein kinase of nuclear factor kappa-B (NF-κB) and c-Jun N-terminal kinases, which are activated by numerous cytokines, growth factors, and microbial products. In this study, we highlighted the functional roles of TAK1 and its interaction with transforming growth factor-β, WNT, AMP-activated protein kinase, and NF-κB signaling pathways in liver inflammation, steatosis, fibrosis, and carcinogenesis based on previously published articles.

## Introduction

Transforming growth factor-β-activated kinase 1 (TAK1) is a member of the mitogen-activated protein (MAP) kinase kinase kinase (MAP3K) (Ajibade et al., 2012; Cohen and Strickson, 2017). This molecule was originally discovered in yeast with the homolog of MAP3K Ste11p in the yeast pheromone-induced MAPK pathway (Yamaguchi et al., 1995). Numerous stimuli, such as interleukin 1 (IL-1), tumor necrosis factor (TNF), transforming growth factor-beta (TGF-β), and toll-like receptor (TLR) ligands, can activate TAK1. Activated TAK1 then stimulates its downstream nuclear factor kappa-B (NF-κB) and c-Jun N-terminal kinases (JNKs). TAK1 has been implicated in embryonic development, inflammation, cell survival, and metabolism (Inokuchi et al., 2010; Mihaly et al., 2014; Mukhopadhyay and Lee, 2020; Xu and Lei, 2020). Recent reports have further determined that TAK1 is involved in autophagy, fatty acid oxidation, steatosis, and carcinogenesis, suggesting that TAK1 is a putative therapeutic target for nonalcoholic steatohepatitis and cancer (Inokuchi et al., 2010; Yang et al., 2013; Inokuchi-Shimizu et al., 2014; Mukhopadhyay and Lee, 2020). Here, we summarize the functional role of TAK1 in liver inflammation, fibrosis, metabolism, and carcinogenesis.

## Regulation of TAK1 Signaling

**FIGURE 1:**
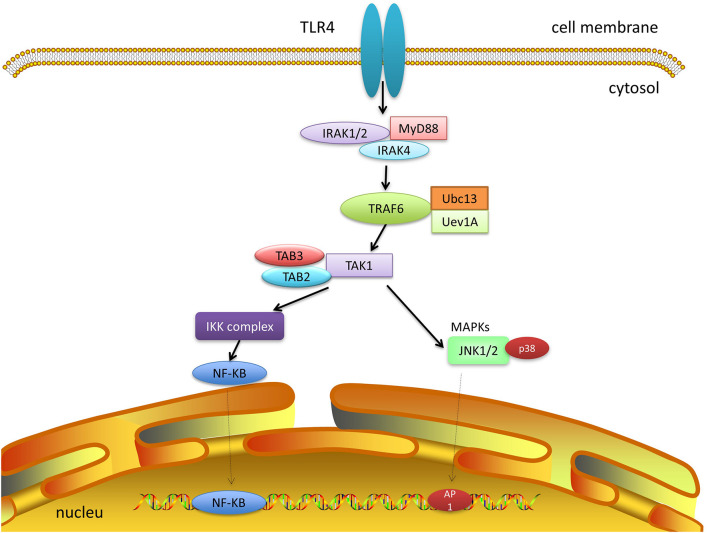
TAK1 signaling. TAK1 signaling is activated after ligand binding to the receptors. TLRs signaling recruits adaptor proteins myD88, which provides a platform for the recruitment of IRAK1/2 and IRAK4. These complexes continually recruit TRAF6, Ubc13, and Uev1A, which bind to TAB2 and TAB3 and lead to the activation of TAK1. Activated TAK1 mediates multiple signal transduction such as MAPK and NF-κB.

A variety of stimuli, such as cytokines, TLR, B-cell and T-cell receptor ligands (Ninomiya-Tsuji et al., 1999; Sakurai, 2012), IL-1β, and TLR4 ligand (lipopolysaccharide [LPS]), if bound to their correlated receptors, can stimulate TAK1, triggering the downstream signaling cascade through the myeloid differentiation primary response protein 88 (MyD88) pathway (Cohen and Strickson, 2017). MyD88 induces interleukin-1 receptor-associated kinase 4 (IRAK4) and then binds by interacting with its specific domains. IRAK1/2 are subsequently activated and detached from MyD88, contributing to the induction of the RING-domain E3 ubiquitin ligase tumor necrosis factor receptor-associated factor 6 (TRAF6). TRAF6, in conjunction with the dimeric E2 ubiquitin-conjugating enzymes (Ubc13) and ubiquitin-conjugating enzyme variant 1A (Uev1A), catalyzes the formation of a Lys63 (K63)-linked polyubiquitin chain on TRAF6 itself, then stimulates a complex of TAK1, TGF-β-activated kinase 1/MAP3K7 binding protein 2 (TAB2), and TAB3. The TAK1 complex promotes the activation of MAPKs, extracellular signal-regulated kinase 1/2(ERK1/2), p38, and JNK, resulting in the activation of activator protein-1 (AP-1) (Figure 1). Simultaneously, the inhibition of nuclear factor-κB (IκB) kinase (IKK) complex, which consists of IKKα, IKKβ, and IKKγ, causes their phosphorylation and ubiquitination, resulting in IκBα dissociation, NF-κB translocation, and full activation (Shibuya et al., 1996; Ninomiya-Tsuji et al., 1999; Wang et al., 2001; Shim et al., 2005; Takeuchi and Akira, 2010; Ajibade et al., 2012; Cohen and Strickson, 2017). During these procedures, TAK1 activation requires the phosphorylation of Thr-184 and Thr-187 residues in the activation loop (Singhirunnusorn et al., 2005; Roh et al., 2014). Recent data revealed that TAK1 activation in mediating downstream signaling requires additional phosphorylation at Ser-412, which discriminates the response of TAK1 to proinflammatory stimuli, such as TNF-α, LPS, and IL-1β (Ouyang et al., 2014).

For the inactivation of TAK1 signaling, recent research revealed that multiple post-translational modifications, such as dephosphorylation and deubiquitylation, are involved in this procedure (Sakurai et al., 2006). Thr-187, the most important autophosphorylation site of TAK1, is dephosphorylated by protein phosphatase 6 (PP6) (Kajino et al., 2006). Then, P38a promotes TAB1 phosphorylation at the Ser-423 and Thr-431 site. Finally, the E3 ubiquitin ligase Itch and deubiquitinase cylindromatosis (CYLD) synergize to cleave the K63-linked polyubiquitin chains from the activated TAK1 and then catalyze the K48-linked ubiquitination at the Lys-72 site to cease the activation of NF-κB mediated by TAK1 (Sakurai et al., 2006; Ahmed et al., 2011; Fan et al., 2012).

## Crosstalk of TAK1 With Other Signaling Pathways

### TAK1 and TGF-β Signaling Pathway

TGF-β is an essential cellular signaling molecule that regulates cell apoptosis, proliferation, differentiation, angiogenesis, embryonic development, inflammation, fibrosis, and carcinogenesis (Batlle and Massague, 2019; Frangogiannis, 2020; Derynck et al., 2021; Gough et al., 2021). TGF-β combines with the receptors (TGF-β receptor 1 and TGF-β receptor 2) and phosphorylates Drosophila melanogaster mothers against decapentaplegic protein 2 (Smad2) and Smad3. To translocate the signal into the nucleus, phosphorylated Smad2/3 bind with Smad4 to form a complex, which plays a vital role in regulating gene transcription (Roh et al., 2014; Batlle and Massague, 2019).

**FIGURE 2:**
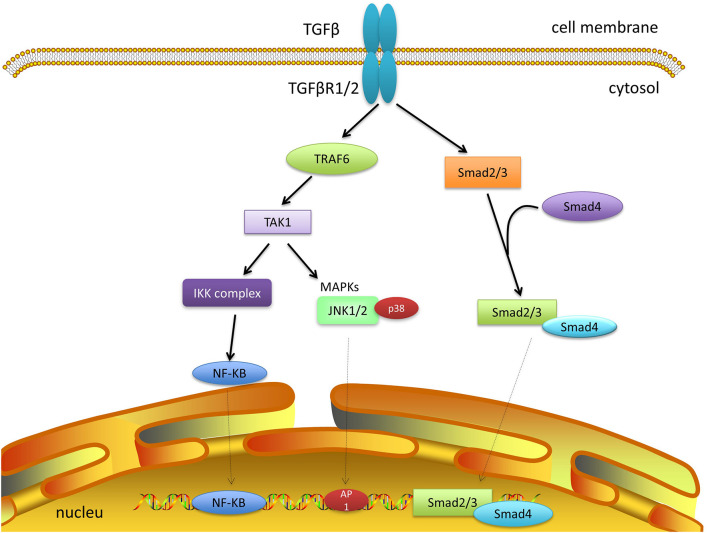
TAK1 and TGF-β signaling. TGF-β and its receptor form a complex and recruit TRAF6. The polyubiquitylation of TRAF6 can activate TAK1, which promotes subsequent cascades, including JNK, P38, and NF-κB. Smad-dependent signaling has not been discussed further here.

TAK1 can rapidly be activated by TGF-β, which can then promote p38 and JNK activation. TRAF6 has been reported to be involved in the mechanism by which TGF-β mediates the activation of TAK1 (Yamaguchi et al., 1995; Sorrentino et al., 2008; Dai et al., 2012). First, TGF-β binds to TGF-β receptors 1 and 2 to form a complex. This complex binds to TRAF6 at the C-terminus, after which TRAF6 is inducted and ubiquitylated. TAK1 on the K34 with K63-linked polyubiquitin chains is also ubiquitylated (Sorrentino et al., 2008; Yamashita et al., 2008; Xia et al., 2009). A recent report revealed that TAK1-mediated activation of IKK, p38, and JNK by TGF-β requires TRAF6 to ubiquitylate K158 on TAK1 (Landstrom, 2010; Mao et al., 2011; Figure 2).

### TAK1 and WNT Signaling Pathway

WNT signaling plays a critical role in cell growth, proliferation, differentiation, and death via the canonical and noncanonical pathways. Deregulation of WNT signaling causes aberrant proliferation and cancer (Dai et al., 2012; Chae and Bothwell, 2018; Bugter et al., 2021; Tewari et al., 2021). In this section, we discuss the most recent and compelling evidence supporting the role of TAK1 in WNT signaling.

**FIGURE 3:**
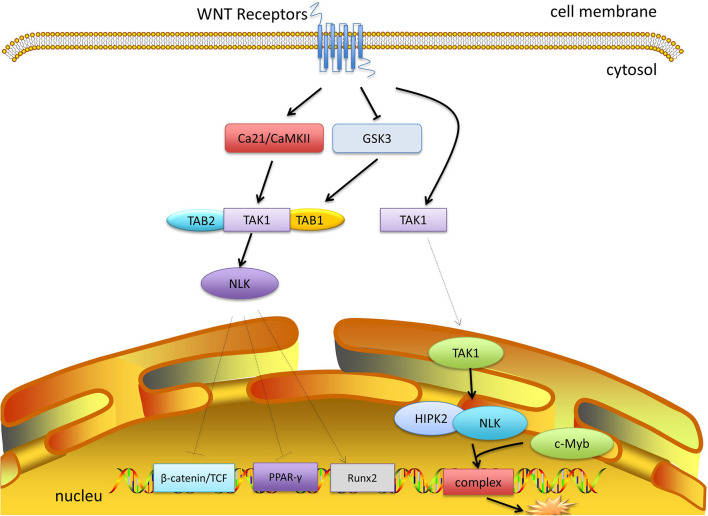
TAK1 and WNT signaling. WNT receptors inhibit the activity of GSK3, which stabilizes the TAB1-TAK1 complex and activates TAK1. WNT-5A signaling can activate Ca21/CaMKII, which promotes the activation of TAK1 and NLK. NLK inhibits the transcriptional activation of β-catenin and TCF, and inhibits PPAR-γ, and activates Runx2. Moreover, after moving into the nucleus, TAK1 recruits HIPK2 and NLK, which bind c-Myb to form a complex and mediate its degradation.

TAK1 was first distinguished to function upstream of the nuclear factor NF-κB essential modulator- (NEMO-) like kinase (NLK). Smit et al. (2004) first confirmed that WNT stimulation leads to autophosphorylation and the activation of TAK1 in a TAB1-dependent manner, which then activates the NLK-MAPK cascade. Furthermore, WNT receptors regulate the activity of glycogen synthase kinase-3 (GSK3), which in turn stabilizes the TAB1-TAK1 complex, thus activating TAK1 (Hoeflich et al., 2000). However, the TAK1-NLK-MAPK cascade can also be activated by the non-canonical WNT-5A/Ca^2+^ pathway to counteract canonical β-catenin signaling (Santoro et al., 2017). A WNT-5A-activated Ca21/CaMKII–TAK1–NLK pathway was shown to stimulate NLK and reduce the transcriptional activation mediated by β-catenin and T cell factor (TCF) (Ishitani et al., 1999). The CaMKII-TAK1-TAB2-NLK pathway is also required for suppressing peroxisome proliferator-activated receptor γ (PPAR-γ) transactivation and induction of Runt-related transcription factor 2 (Runx2) expression (Takada et al., 2007). C-Myb plays a discriminating role in immature hematopoietic cell proliferation and the development of early T-cells. TAK1 is involved in the inhibition of c-Myb mediated by WNT1 (Dai et al., 2012). The WNT1 signal initially induces the translocation of TAK1 to the nucleus, which then activates homeodomain-interacting protein kinase 2 (HIPK2) and NLK. NLK, HIPK2, and c-Myb bind together to form a complex and phosphorylate c-Myb at multiple sites, followed by the degradation of c-Myb through ubiquitination and proteasome-dependent pathways (Kanei-Ishii et al., 2004). TAK1 also contributes to the inhibition of a-Myb mediated by WNT1 (Kurahashi et al., 2005). Recent data demonstrated that TAK1 is required for TGF-β-induced WNT-5A expression. Regulated by TAK1, β-catenin is a critical factor in the induction of WNT-5A. In this process, TGF-β activates TAK1, which then induces p38 and JNK to recruit WNT-5A and Sp1 (Kumawat et al., 2014). However, it is still unclear whether E3 ubiquitin ligase is involved in the interaction of the WNT and TAK1 pathways (Figure 3).

### TAK1 and AMP-Activated Protein Kinase Signaling Pathway

AMP-activated protein kinase (AMPK), which is a heterotrimeric protein, contains a catalytic subunit (α) and two regulatory subunits (β and γ) (Ross et al., 2016; Gonzalez et al., 2020). AMPK functions as an energy and nutrient status sensor in the cell (Hardie et al., 2012, 2016; Hardie, 2018; Herzig and Shaw, 2018; Lin and Hardie, 2018). The activation of AMPK is typically mediated by an increase in the adenosine monophosphate/adenosine triphosphate (AMP/ATP) ratio. Subsequently, activated AMPK regulates energy metabolism by stimulating ATP producing pathways and inhibiting energy consuming pathways (Carling et al., 2011).

**FIGURE 4:**
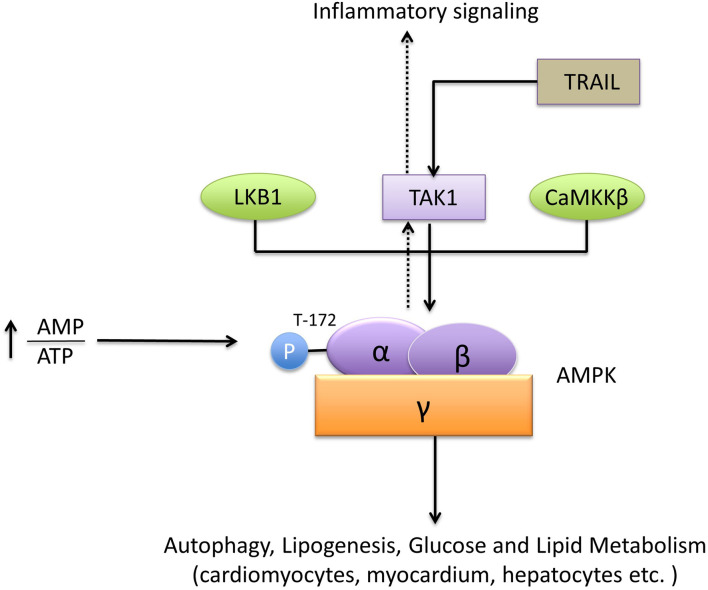
TAK1 and AMPK signaling. AMPK is a heterotrimeric protein formed by the subunits α, β, and γ. The recognized upstream proteins of AMPK include LKB1, CaMKKβ, and TAK1. TAK1 mediates the phosphorylation of AMPK at T172 and promotes its activation. The downstream pathways, including autophagy, lipogenesis, glucose, and lipid metabolism, are subsequently activated by AMPK.

TAK1 was identified as a functional member of the sucrose non-fermenting 1 (Snf1) /AMPK kinase family in mammalian cells (Momcilovic et al., 2006). TAK1 is required for the phosphorylation of AMPK at T172, which induces AMPK activation. TAK1 activation also promotes AMPK substrate acetyl-CoA carboxylase phosphorylation in cardiomyocytes and myocardium *in vitro*. In this process, endogenous TAK1 is essential for the activation of AMPK kinase, Live kinase B1, and calmodulin-dependent protein kinase kinase beta, which triggers AMPK activity (Xie et al., 2006; Scholz et al., 2010). Tumor necrosis factor (TNF)-related apoptosis-inducing ligand (TRAIL) activates the AMPK pathway through TAK1 to modulate autophagy; however, the underlying mechanism is unknown (Herrero-Martin et al., 2009). Interestingly, in T cells, AMPK-a1 was identified as an activating kinase of TAK1, mediating inflammatory signals triggered by TLR4 and TNF-α. Whereas, the studies in hepatocytes indicated that TAK1 was the upstream molecule of AMPK and played a central role in lipogenesis and autophagy (Kim et al., 2012; Inokuchi-Shimizu et al., 2014). Therefore, the physiological role of TAK1/AMPK or AMPK/TAK1 signals in different cell types is inconsistent and remains uncertain (Neumann, 2018; Figure 4).

### TAK1 and CARD-BCL10-MALT1 Signaling Pathway

The caspase activation and recruitment domain (CARD) protein, B-cell lymphoma 10 (BCL10), and mucosa-associated lymphoid tissue lymphoma translocation protein 1 (MALT1) comprise the CARD-BCL10-MALT1 (CBM) complex, which is a key signaling node that mainly mediates immune responses such as T-cell or B-cell receptor-dependent lymphocyte activation. There are four types of presently known CBM complexes. The main difference between them is the CARD protein, which can be composed of either CARD9, CARD10 (CARMA3), CARD 11 (CARMA1), or CARD14 (CARMA2) (Ruland and Hartjes, 2019).

**FIGURE 5:**
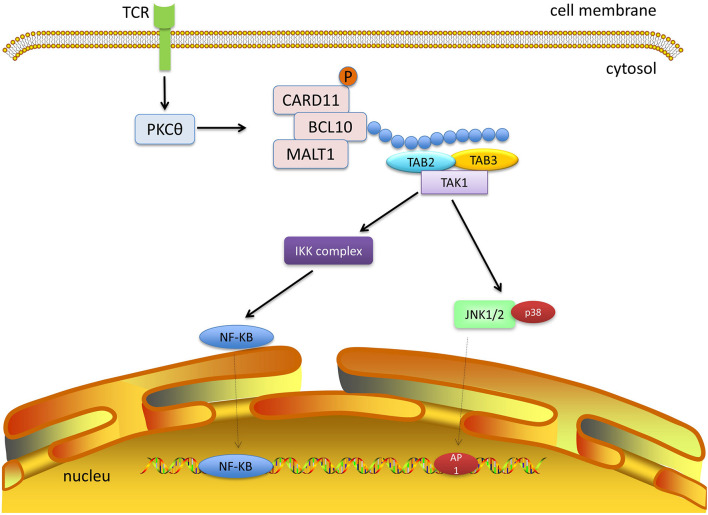
TAK1 and CBM signaling. The protein kinase Cθ (PKCθ) is activated after stimulation of the T-cell receptor. PKCθ phosphorylates CARD11, which subsequently binds to BCL10 and MALT1. The CBM complex recruits E3 ubiquitin ligases to form ubiquitin chains, which provide platforms for TAB2 and TAB3 and recruit TAK1. Next, TAK1 mediates activation of the NF-κB and MAPK pathways.

TAK1 can be activated by the CBM complex. The well-studied CBM signaling pathway is the CARD11-BCL10-MALT1 pathway in T lymphocytes (Figure 5). Upon stimulation of the T-cell receptor, the protein kinase Cθ (PKCθ) is activated through a series of steps (Brownlie and Zamoyska, 2013). PKCθ can phosphorylate CARD11, which oligomerizes BCL10 and MALT1 to form filamentous complexes (Matsumoto et al., 2005; Sommer et al., 2005). The CBM complex recruits E3 ubiquitin ligases, such as TRAF6, cellular inhibitor of apoptosis 1 (cIAP1), cIAP2, and linear ubiquitin chain assembly complex. All the enzymes can facilitate the ubiquitination of CBM. The ubiquitin chains provide docking platforms for TAB2 and TAB3, and the recruitment of TAK1, which in turn mediates the activation of NF-κB and MAPK pathways (Sun et al., 2004). The stimulation of other types of CARDs is similar to CARD11, and different CARDs can be activated by different receptor signals. For instance, CARD9 is often activated by dectin1 or dectin2 (Gross et al., 2006; Robinson et al., 2009). Epidermal growth factor receptor (EGFR) and G protein coupled receptor (GPCR) can induce the activation of CARD10 (McAllister-Lucas et al., 2007). And dectin1 can also stimulate CARD14 (Schmitt et al., 2016). The activated CARDs combine with BCL10 and MALT to form the CBM complexes. The highly homologous CBM complexes can recruit TAK1 and trigger NF-κB and MAPK signaling pathways by ubiquitin ligases, and eventually promote the expression of inflammation-related genes.

### TAK1 and Other Signaling Pathways

**FIGURE 6:**
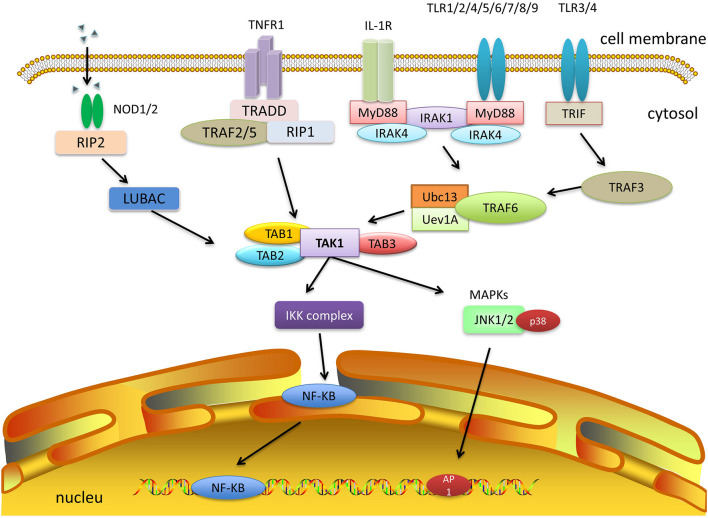
TAK1 and other signaling pathways. IL-1, TNF-α, TLR, and NLR signaling pathways can activate TAK1 and downstream cascades. Among them, both IL-1 and TLR signaling pathways need to activate TRAF6, which combines with Ubc13/Uev1a, and then activates TAK1. TNF-α signaling recruits TRAF2 or TRAF5 and promotes the polyubiquitination of RIP1 to facilitate the activation of TAK1. NLR signaling recruits TAK1 and TABs via autophosphorylation of RIPK2 and its interaction with LUBAC. The formation of a complex of TAK1 and TABs will eventually trigger either the NF-κB or MAPK pathways.

IL-1, TNF-α, and TLR signaling pathways, which mediate the transcription of the pro-inflammatory and NOD-like receptor (NLR) families, all require the activation of TAK1, which suggests that it holds the core position in the signaling cascades and is a druggable target that can reduce the overall inflammatory response in some chronic diseases. A summary of each pathway is provided below (Figure 6).

Both IL-1 and TLR signaling pathways need to activate TRAF6. After ligand binding to the receptor, TLR family members (TLR1/2/4/5/6, TLR7/8/9) and some IL-1R superfamily members (IL-1/18/33R) initiate MyD88 recruitment, bind and activate IL-1 receptor-associated kinase 1 or 4 (IRAK1/4), and thereby activate TRAF6. Furthermore, other TLR family members (TLR3/4) can also be linked by TIR-domain-containing adaptor inducing interferon-β (TRIF), which recruits TRAF3 and activates TRAF6 (Hacker et al., 2006, 2011; Kawai and Akira, 2011; Cohen, 2014; Kawasaki and Kawai, 2014). Upon activation, TRAF6 can form a complex with the ubiquitin-conjugating enzyme Ubc13 and its variant Uev1a, conjugating K63-linked polyubiquitin chains to transduce the signal (Ea et al., 2004; Yang et al., 2004). The C-terminal Npl4 zinc finger domain of TAB2 and TAB3 binds to the polyubiquitin chains of TRAF6, resulting in the activation of TAK1 (Hirata et al., 2017).

As for TNF-α signaling, once TNF-α binds to TNF receptor 1 (TNFR1), the adaptor molecule TNFR1-associated DEATH domain protein (TRADD) is recruited by the TNF receptor 1, which in turn recruits TRAF2 or TRAF5. However, neither TRAF2 nor TRAF5 was able to activate TAK1 directly. Instead, they promote K63-linked polyubiquitination of receptor-interacting protein 1 (RIP1) (Adhikari et al., 2007). Subsequently, the polyubiquitin chains of RIP1 bind to the C-terminal Npl4 zinc finger domain of TAB2 and TAB3, which allows the autophosphorylation-dependent activation of TAK1 to occur after conformational changes (Wang et al., 2001; Kanayama et al., 2004; Lee et al., 2004).

Despite the different types of TRAFs that can be activated, they all result in TAB activation, which recruits TAK1 and forms the TAB-TAK-TRAF complex. TAK1 is then, mono- and polyubiquitinated at Lys34 (Hamidi et al., 2012). Upon ubiquitination, TAK1 phosphorylates its downstream targets and activates the MAPK and NF-κB pathways, which in turn lead to the production of inflammatory cytokines.

In addition, the NLR family also plays fundamental and pleiotropic roles in inflammation and host defense against infection (Mukherjee et al., 2019). Active nucleotide-binding oligomerization domain 1 and 2 (NOD1/2) multimers stimulate autophosphorylation of RIP2 kinase (also known as RIPK2), RIP2 then mediates the recruitment of TAK1, TAB1, TAB2, or TAB3 (Wang et al., 2001; Chen et al., 2006; Liu et al., 2019), which subsequently activates downstream NF-κB and MAPK pathways.

## TAK1 and Function

### TAK1 and Cell Death

The stimulation of exogenous and endogenous ligands, such as microbial products, cytokines, growth factors, TNF-α, and TRAIL can activate TAK1 and subsequent cell death and survival signals (Shim et al., 2005; Mihaly et al., 2014; Ogura et al., 2015). Nuclear translocation of NF-κB is essential for TAK1 mediated cell survival. TAK1 deficiency cannot activate NF-κB upon treatment with TNF-α or TGF-β and promotes keratinocyte or hepatocyte apoptosis (Inokuchi et al., 2010; Roh et al., 2014). There are two distinct pathways of TAK1 that protect cells from TNF-α-induced apoptosis (Mihaly et al., 2014). On one hand, NF-κB can inhibit the activation of caspase by upregulating antiapoptotic proteins such as cellular FADD-like IL-1beta-converting enzyme-inhibitory protein (Irmler et al., 1997; Chang et al., 2006; Oberst and Green, 2011) (similar to the structure of caspase-8 but without protease activity) and the inhibitor of apoptosis proteins (IAP family including cIAP1, cIAP2, and XIAP) (Deveraux et al., 1998; Wang et al., 1998). While on the other hand, the accumulation of TNF-α-induced reactive oxygen species (ROS) in the cells leads to the depletion of cIAP proteins (Morioka et al., 2009), and the degradation products of cIAP lead to the deubiquitylation of receptor-interacting protein kinase 1 (RIPK1), which eventually activates caspase-8 in a kinase-dependent manner (Ofengeim and Yuan, 2013). However, TAK1 inhibits ROS production and thus prevents apoptotic cell death.

Furthermore, it has been reported that TAK1 participates in the RIPK1- and RIPK3- mediated necroptosis pathway, despite its pro-survival function. The prolonged hyperactivation of TAK1 can induce necrosis which is mediated by TNF-α through the phosphorylation and activation of RIPK3, which further activates TAK1 and forms a positive feedback loop of RIPK1, RIPK3, and TAK1 (Morioka et al., 2014). RIPK3 phosphorylates mixed lineage kinase domain-like (MLKL), which induces its oligomerization, resulting in rupture of the plasma membrane (Sun et al., 2012; Cai et al., 2014; Wang et al., 2014). These results confirm that TAK1 is an essential regulator of TNF-mediated cell death.

TAK1 also controls TGF-β-mediated hepatocyte death, hepatocytes with TAK1 deficiency were susceptible to cell death mediated by TGF-β when there was enhanced phosphorylation of Smad2/3 and inactivation of NF-κB, while inhibition of the Smad pathway with siRNA-Smad2 alleviated TGF-β induced hepatocyte death (Yang et al., 2013). These reports demonstrate that TAK1 promotes the NF-κB pathway for cell survival and inhibits the over-activation of TGF-β-mediated Smad2/3, which promotes cell death in hepatocytes. In addition, the function of TAK1 in the cell death of hepatocytes, with lipid accumulation in response to TGF-β, has also been studied. When using 5z-7-oxozeaenol (a TAK1 inhibitor) to block the activity of TAK1, cell death of hepatocytes treated with TGF-β increased, and cell death of both palmitate and TGF-β-treated hepatocytes further increased. These findings indicate that TAK1 has a negative effect on the regulation of TGF-β-induced apoptosis in steatotic hepatocytes (Yang et al., 2014).

Similarly, the TAK1/JNK pathway is regulated by the cellular repressor of E1A-stimulated genes (Creg). Deletion of hepatocyte-specific Creg promotes cell death, suppresses cell proliferation, and worsens inflammatory responses during liver ischemia/reperfusion injury. While using a specific TAK1 inhibitor, 5z-7-oxozeaenol, in Creg^ΔHEP^ mice before ischemia/reperfusion injury, abolished the activation of phosphorylation of JNK, P38, and NF-κB, reduced cell death, and improved the damage to liver function and cell proliferation (Yang et al., 2019). Thus, TAK1 participates in MAPK-induced hepatocyte inflammation and cell death.

### TAK1 and Liver Fibrosis

The deletion of TAK1 in hepatocytes leads to fibrosis (Song et al., 2018). TAK1^ΔHEP^ mice started to present liver fibrosis at the age of 1 month, and the extent of fibrosis further increased at 4 months of age. No gender differences in liver fibrosis were observed in TAK1^ΔHEP^ mice. The number of activated hepatic stellate cells (HSCs) and levels of fibrogenic parameters were drastically increased in TAK1^ΔHEP^ mice compared to those in wild-type mice (Inokuchi et al., 2010). In addition, it was found that the level of TGF-β1 in Kupffer cell-depleted TAK1^ΔHEP^ mice was lower than that in the control TAK1^ΔHEP^ mice, which suggests that TGF-β1 is mainly produced by Kupffer cells in TAK1^ΔHEP^ mice. These findings demonstrate that liver injury in TAK1^ΔHEP^ mice is related to inflammation and TGF-β1 originating from Kupffer cells induces the activation of HSCs, resulting in liver fibrosis.

Subsequently, the role of TGF-β signaling in the progression of liver fibrosis in TAK1^ΔHEP^ mice was further explored. Interestingly, scientists found that TAK1/Tgfbr2^ΔHEP^ mice developed less spontaneous liver fibrosis as compared to the control TAK1^ΔHEP^ mice. The number of activated HSCs and the production of fibrogenic genes were drastically downregulated in TAK1/Tgfbr2^ΔHEP^ mice compared to that in the control TAK1^ΔHEP^ mice. These findings suggest that TGF-β signaling in hepatocytes is essential for the development of spontaneous liver fibrosis in TAK1^ΔHEP^ mice (Yang et al., 2013).

Hepatic stellate cells are the major cell type responsible for extracellular matrix synthesis during liver fibrogenesis (Friedman, 2000). After a fibrogenic stimulus, HSCs change into proliferating cells with α-smooth muscle actin (α-SMA) expression and collagen production, which is partially mediated by intracellular kinases (Maher et al., 1988; Maher and McGuire, 1990). Suppression of TAK1 in HSCs decreased the expression of α-SMA protein and HSC proliferation (Schnabl et al., 2001). These results indicate that TAK1 plays a crucial role in HSC activation. Cell-specific inhibition of TAK1, in HSCs in a liver fibrosis model *in vivo*, will be necessary to fully define the contribution of TAK1 in liver fibrosis.

### TAK1 and Lipid Metabolism

The function of TAK1 during lipid metabolism in hepatocytes has also been reported. Peroxisome proliferator-activated receptor α (PPARα), which targets genes and β-oxidation, thus regulates hepatic lipid degradation, was also suppressed in TAK1-deficient hepatocytes. Upon intake of a high-fat diet, TAK1 activity prevented excessive lipid accumulation, cell injury, and inflammation in hepatocytes. These findings suggest that TAK1 modulates lipid metabolism (Inokuchi-Shimizu et al., 2014).

Kang et al. (2011) also analyzed the role of TAK1 in hepatic steatosis. However, they used TAK1^–/–^ mice instead of TAK1^ΔHEP^ mice and had very different findings. They discovered that one-year-old TAK1^–/–^ mice did not suffer from age-induced hepatic steatosis, and TAK1^–/–^ mice were found to be protected against high-fat diet-induced hepatic steatosis in comparison with the wild-type mice. One-year-old male TAK1^–/–^ mice and TAK1^–/–^ mice who were fed a high-fat diet for 6 weeks gained less weight and smaller epididymal and abdominal white adipose tissue as compared to the wild-type mice. The levels of triglyceride, total cholesterol, low-density lipoprotein, and glucose were drastically reduced in the TAK1^–/–^ mice compared with wild-type littermates. These results may be because TAK1 positively modulates the levels of several gene encoding proteins associated with lipid uptake and triglyceride accumulation (Kang et al., 2011).

A recent study (Morioka et al., 2016) has confirmed that sterol regulatory element-binding proteins (SREBPs), a series of key transcription factors that maintain lipid homeostasis and regulate cholesterol and fatty acid biosynthesis, were regulated by TAK1. Compared to the control group, the binding activity of SREBP DNA and the expression of the SREBP gene were increased in the TAK1^LKO^ liver. When TAK1^LKO^ mice were treated with fatostatin, an inhibitor of SREBP, they had lower levels of blood triglycerides and cholesterol. Moreover, they also revealed that TAK1 phosphorylated SREBP in an *in vitro* model, and the ability of SREBP to bind to the sterol-responsive element DNA sequence was enhanced in the TAK1-deficient liver. Hence, one possible explanation for the TAK1 regulation of SREBPs is that the TAK1-phosphorylated SREBPs possess diminished transcriptional activity due to their reduced ability to bind DNA. Further investigations are needed to determine the mechanism underlying the regulation of TAK1 on SREBPs.

In addition, numerous studies have revealed the role of upstream molecules in hepatic steatosis by directly or indirectly regulating TAK1, which may be potential targets for improving lipid metabolism and reducing fat deposition. Some molecules, such as TNFAIP3 interacting protein 3 (TNIP3) (Liu et al., 2020), tumor necrosis factor-α-induced protein 8-like 2 (TIPE2) (Liu et al., 2021), the deubiquitinating enzyme cylindromatosis (CYLD) (Ji et al., 2018), the regulator of G protein signaling (RGSs) proteins (Wang et al., 2021), dual-specificity phosphatase 14 or 26 (Dusp14/Dusp26) (Wang et al., 2018; Ye et al., 2019), and ubiquitin-specific protease 4 or 18 (USP4/USP18) (An et al., 2017; Zhao et al., 2018), can alleviate metabolic disturbances or hepatic inflammation by suppressing TAK1 activation and its downstream signaling cascade of JNK/p38. Other molecules, including the E3 ligase tripartite motif 8 (TRIM8) (Yan et al., 2017) and tumor necrosis factor receptor-associated factor 3 (TRAF3) (Wang et al., 2016) can promote liver steatosis and insulin resistance by activating the phosphorylation of TAK1 and its downstream JNK/p38 and NF-κB signaling. These findings will enhance our understanding of the molecular link and signaling crosstalk between lipid metabolism and non-alcoholic fatty liver disease (NAFLD). We believe that the upstream molecules that inhibit TAK1-JNK/p38 signaling specifically in the liver, show considerable promise as potential drugs for the treatment of NAFLD or metabolic syndrome.

### TAK1 and Hepatic Carcinogenesis

TAK1 deficiency in hepatocytes contributes to the occurrence and progression of hepatocellular carcinoma (Roh et al., 2014; Krishna-Subramanian et al., 2019). Inokuchi-Shimizu et al. (2014) found that liver tumors could be discovered as early as 4 months in TAK1^ΔHEP^ mice, and liver tumor nodules were detected in 80% of the 9-month-old TAK1^ΔHEP^ mice. In contrast, no tumor nodules were found in 12-month-old wild-type mice. Moreover, the expression of α-fetoprotein drastically increased in tumors found in TAK1^ΔHEP^ mice compared to that in wild-type mice. These results indicate that TAK1^ΔHEP^ mice spontaneously develop liver tumors (Inokuchi et al., 2010). In addition, a group from Germany, conditionally ablated TAK1 in liver parenchymal cells (TAK1LPC-KO mice) and reported that they reproduced hepatocyte dysplasia and carcinogenesis (Bettermann et al., 2010). Moreover, Tey et al. (2017) revealed that nuclear Met promotes hepatocellular carcinoma tumorigenesis and metastasis by upregulating TAK1 and activating the NF-κB pathway. Specifically, nuclear Met overexpression promotes the transcription of TAK1, which phosphorylates IKKβ, leading to IκBα phosphorylation and subsequent proteasomal degradation. The released NF-κB activates pro-metastatic genes responsible for HCC cell migration, invasion, tumorigenesis, and metastasis. NF-κB, downstream of TAK1, plays a critical role in innate immunity, inflammation, cell death, and carcinogenesis. NF-κB activation is coordinated by the IKK complex. The double deletion of TAK1 and the nuclear factor NF-κB essential modulator (NEMO) attenuated hepatocyte necrosis, hyperplasia, hepatocarcinogenesis, and cholestasis in TAK1LPC-KO mice (Bettermann et al., 2010). Consequently, TAK1 might potentially mediate carcinogenic responses in the presence of NEMO (Bettermann et al., 2010).

In addition to NEMO, the role of TGF-β signaling in TAK1^ΔHEP^ mice has been investigated in liver cancer. The levels of TGF-βR2, phosphorylated Smad2/3, and the genes associated with the TGF-β signaling pathway were significantly increased in hepatocellular carcinoma (HCC) lesions of TAK1^ΔHEP^ mice compared with wild-type normal livers. These findings indicate that TGF-β signaling activation is related to the development of spontaneous hepatocarcinogenesis in TAK1^ΔHEP^ mice. Scientists have investigated the function of TGF-β signaling in the progression of spontaneous HCC in TAK1^ΔHEP^ mice and found that spontaneous hepatocarcinogenesis was found in 9-month-old TAK1^ΔHEP^ mice. In contrast, TAK1/Tgfbr2^ΔHEP^ mice developed significantly smaller and fewer tumors when compared with TAK1^ΔHEP^ mice. These findings suggest that TGF-β signaling is responsible for the development of spontaneous hepatocarcinogenesis in TAK1^ΔHEP^ mice (Yang et al., 2013).

Furthermore, Inokuchi-Shimizu et al. (2014) also found that excessive mechanistic target of rapamycin complex (mTORC1) activity and the impression of autophagy in TAK1^ΔHEP^ mice are responsible for spontaneous HCC. The inhibition of mTORC1, in turn, inhibited hepatocyte cell death and reduced serum alanine aminotransferase levels in TAK1^ΔHEP^ mice. Additionally, inhibition of mTORC1 activity and restoration of autophagy protected mice from hepatocarcinogenesis. Early-stage treatment drastically downregulated the number of tumors, although there was no change seen in their maximal size, in TAK1^ΔHEP^ mice. Furthermore, late treatment notably reduced the size and number of liver tumors in TAK1^ΔHEP^ mice (Inokuchi-Shimizu et al., 2014).

## Conclusion

TAK1 is a pivotal mediator of innate immunity that can be activated by many factors, including pathogen-associated molecular patterns or damage-associated molecular patterns. TAK1 mediates the activation of downstream signaling pathways, including the NF-κB, p38, JNK, and extracellular signal-regulated kinase pathways, through ubiquitination and phosphorylation. TAK1, the central hub for diverse signaling pathways, interacts with TGF-β and WNT signaling to regulate cell death or carcinogenesis and connects with IL-1, TNF-α, TLR, and NLR signaling in the host immune defense response. Furthermore, TAK1 physiologically regulates AMPK signaling and autophagy and negatively regulates mTORC1. The accumulation of evidence indicated that TAK1 is essential for liver inflammation, lipid metabolism, fibrosis, and hepatocarcinogenesis.

However, existing studies indicate that TAK1 plays a distinct role in different animal models. For example, mice with hepatocyte-specific TAK1 deletion often present lipid accumulation, hepatocyte injury, and liver inflammation. Thus, TAK1 seems to play a protective role in the liver, however, opposite phenotypes were observed in mice with TAK1 complete knock-out, which showed lower hepatic triglyceride levels and reduced lipid accumulation in adipose tissue. Thus, TAK1 appears to be harmful to organisms. These seemingly contradictory results may be due to the opposite roles of TAK1 in regulating metabolism in the liver and other organs such as the brain, heart, kidney, and adipose tissue. On one hand, TAK1 outside the liver may promote adipogenesis or inhibit lipolysis. While on the other hand, the overall equation that supports the balance between the energy coming in and going out, such as the amount of exercise or food intake, may be influenced by the regulation of TAK1. However, research on the effects of TAK1 on other organs is limited. A previous study (Cui et al., 2014) indicated that cardiomyocyte-specific deletion of TAK1 could promote apoptotic and necrotic cell death in the heart. Selective deletion of TAK1 in the myeloid cells of mice leads to splenomegaly (Ajibade et al., 2012), lymphadenopathy, and apoptosis in the macrophages. Inactivation of TAK1 in the satellite cells of mice can inhibit muscle regeneration (Ogura et al., 2015). Whether these processes are linked to lipid metabolism and energy consumption is inconclusive. In addition, the role of TAK1 in the liver seems to be sophisticated and paradoxical. TAK1 can protect mice against lipid accumulation, hepatosteatosis, hepatic injury, and inflammation (Inokuchi-Shimizu et al., 2014). However, many studies suggest that the inhibition of the TAK1 signaling pathway could alleviate the progression of NAFLD (Wang et al., 2016, 2018, 2021; An et al., 2017; Yan et al., 2017; Ji et al., 2018; Zhao et al., 2018; Ye et al., 2019; Liu et al., 2020, 2021). The fundamental mechanism for the seemingly contradictory conclusions needs further investigation in different cell types.

In conclusion, the role of TAK1 in different cells, genetically engineered mouse models, and different signaling pathways are distinct. Considering the intricate role of TAK1 and the complex signaling pathway network, direct targeting of TAK1 itself without organ and environmental specificity may have unpredictable consequences. Because of its central role in above-mentioned pathways, it will be potential therapeutic target to use small molecular compound to aim at the upstream or downstream of its key regulatory signaling pathways in specific cells and specific environments.

## Author Contributions

WW and WG searched the literature and drafted the manuscript. QZ determined and refined the structure. AA checked and polished syntax and grammar. LY and ES critically revised the important intellectual content of this manuscript and approved the work. All authors contributed to the article and have read and agreed to the final submitted version.

## Conflict of Interest

The authors declare that the research was conducted in the absence of any commercial or financial relationships that could be construed as a potential conflict of interest.

## Publisher’s Note

All claims expressed in this article are solely those of the authors and do not necessarily represent those of their affiliated organizations, or those of the publisher, the editors and the reviewers. Any product that may be evaluated in this article, or claim that may be made by its manufacturer, is not guaranteed or endorsed by the publisher.

## Abbreviations

AMP: adenosine monophosphate
ATP: adenosine triphosphate
CaMKII: Ca2+/calmodulin-dependent protein kinase II
CTGF: connective tissue growth factor
DAMP: danger-associated molecular pattern
HIPK: homeodomain-interacting protein kinase
IKK: IκB kinase
IL-1: interleukin-1
IRAK: IL-1 receptor-associated kinase
JNK: c-JunN-terminal kinase
LPS: lipopolysaccharide
LSEC: liver sinusoidal endothelial cells
mTORC: mammalian target of rapamycin complex
MyD88: myeloid differentiation factor 88
NEMO: NF-κB essential modulator
NF-κB: nuclear factor κB
NLK: Nemo-like kinase
PI3K: phosphatidylinositol 3-kinase
PPAR: peroxisome proliferator-activated receptor
RING: really interesting new gene
RIPK: receptor-interacting protein kinase
SMA: smooth muscle actin
TAB: transforming growth factor [TGF]-β-activated kinase 1/mitogen-activated protein kinase kinase kinase 7 [MAP3K7]-binding protein
TCF: T cell factor
TGF-β: transforming growth factor-β
TNF: tumor necrosis factor
TRAF: tumor necrosis factor receptor-associated factor
TRAIL: tumor necrosis factor-related apoptosis-inducing ligand
VEGF: vascular endothelial growth factor.
